# Navigating the self online

**DOI:** 10.3389/fpsyg.2025.1499039

**Published:** 2025-03-05

**Authors:** Shisei Tei, Junya Fujino, Toshiya Murai

**Affiliations:** ^1^Department of Psychiatry, Graduate School of Medicine, Kyoto University, Kyoto, Japan; ^2^Department of Psychiatry and Behavioral Sciences, Graduate School of Medical and Dental Sciences, Tokyo Medical and Dental University, Tokyo, Japan

**Keywords:** self, narrative, digital era, embodiment, autopoiesis, bodily experience, default mode network, public mental health

## Introduction

Rapid digitization is reshaping our daily lives and worldview. Driven by advancements in information and communication technology (ICT) and artificial intelligence (AI), this change affects our cognition and behavioral patterns. These technologies facilitated remote work through online connections (i.e., mediated communication; Supplementary Table S1), which became particularly crucial during crises like the COVID-19 pandemic. However, while digitization offers numerous benefits, it also poses challenges (Cataldo et al., 2022).

Problematic online communications and AI-human interactions can distort narrative aspect of self, affecting how we perceive ourselves and connect with others. Our natural inclination to interpret sequences of experiences as narratives poses particular risks in digital environments, often manifesting in behaviors such as excessive smartphone use or overreliance on the internet (Carr, 2010; Gritti et al., 2023; McAdams, 2001). This tendency can lead to the formation of extreme beliefs, intergroup conflicts, and altered senses of belonging (Jackson, 2023; Jaworsky and Qiaoan, 2021; Tei and Fujino, 2022).

Advancements in ICT have affected self-perception in both beneficial and detrimental ways. While increased self-awareness through these technologies can enhance social interactions, it can also lead to negative phenomena such as *Zoom fatigue*, which impacts personal presence and hinders effective social attunement (Shteynberg et al., 2023; James et al., 2023). Virtual platforms, including Zoom, have redefined the dynamics of face-to-face interactions, leading to heightened self-awareness during video calls. In these settings, participants often monitor their own image and interpret others' emotions and intentions based on limited bodily cues such as eye contact or facial expressions (Osler and Zahavi, 2023).

This commentary investigates how self-perception is shaped by online and in-person interactions. By linking narrative and embodied theories with relevant cognitive studies such as online self-externalization, we aim to deepen our understanding of the ways digital environments influence our sense of self.

## Impact of communication technology on self-perception

Online interactions can alter self-perception both subjectively (from the individual's perspective) and objectively (from an external observer's perspective), bringing the self into focus within the recreated digital realms. These effects are often exacerbated by hyper-customization in social media, which can alter social behaviors, reinforce the construction of an idealized self-image, and increase sensitivity to others' perceptions (Cataldo et al., 2022; Finlayson-Short et al., 2021; Tei and Wu, 2021). This sensitivity encompasses how individuals imagine they are perceived and evaluated by others, reflecting social and cognitive alterations (Bolis et al., 2023; Tei et al., 2020).

Specifically, altered self-perception is observed in phenomena associated with external affirmation or online popularity, such as the *fear of missing out* (FOMO), where individuals experience anxiety about exclusion from social events (Cataldo et al., 2022). Other behaviors include excessive engagement in activities such as taking *selfies* and experiencing the *echo chamber effect*, where one is only exposed to information that reinforces their existing beliefs. Another trend is *fitspiration*, where individuals pursue unrealistic body ideals and fitness goals often portrayed on social media. Furthermore, identity distortion may also occur when individuals create an online persona (Turkle, 2011), reshaping their self-narrative.

## The self as a center of narrative gravity

The narrative theory suggests that the self acts as the gravitational center of life stories (Dennett, 2014), emphasizing its dynamic nature rather than a fixed entity (e.g., Zhang et al., 2024). According to this theory, individuals serve as focal points around which their narratives, experiences, and perceptions revolve. Just as gravity pulls objects toward the center of a mass, narratives unify diverse life experiences into a coherent sense of self. In this way, shaped by these experiences, the self becomes the center of narrative gravity.

This narrative theory extends into an interpersonal context, emphasizing how narratives shape both individual identities and collective worldviews. The center of narrative gravity represents the core of social experiences, allowing for the continuous updating of shared narratives. This suggests that experiences, interpretations, and stories are not solely personal endeavors but are co-created within larger social groups.

## Online communications and the narrative self

The perception of self can emerge as a dynamic narrative gravity center in both online and in-person communication, but the fluidity of the digital environment enhances self-understanding at the cost of stability (Heersmink, 2017, 2020; Zhang et al., 2024). It exposes the narrative self to greater vulnerability from feedback (Turkle, 2011; Agai, 2022; Ashuri et al., 2018), as social interactions crystallize through posts and digital footprints.

More specifically, online communications can enhance and hinder our narrative (Bortolan, 2024). The rapid pace and emotional intensity of virtual interactions amplify self-presentation and affirmations, fostering connection (Floridi, 2011; Toma and Hancock, 2013). However, the curated nature of social media, such as *likes* and comments, can distort the narrative self and increase interpersonal anxiety (Fineberg et al., 2022). This dual impact reinforces the narrative self as a protagonist while promoting instability in self-perception (McAdams, 2001; Turkle, 2011; Heersmink, 2020). Thus, engaging with multiple digital platforms can enrich self-narratives but also risk fragmentation, especially among adolescents and psychiatric populations (Aboujaoude, 2010, 2011; Heersmink, 2017; Turkle, 1999). In addition, although virtual anonymity reduces social pressure and encourages identity exploration, it can create tensions between online and offline selves (Bargh et al., 2002), potentially leading to superficial self-narratives and a disconnection from the body, closely tied to online self-externalization discussed next.

## Self-externalization, narrative and embodied cognition

**Figure 1:**
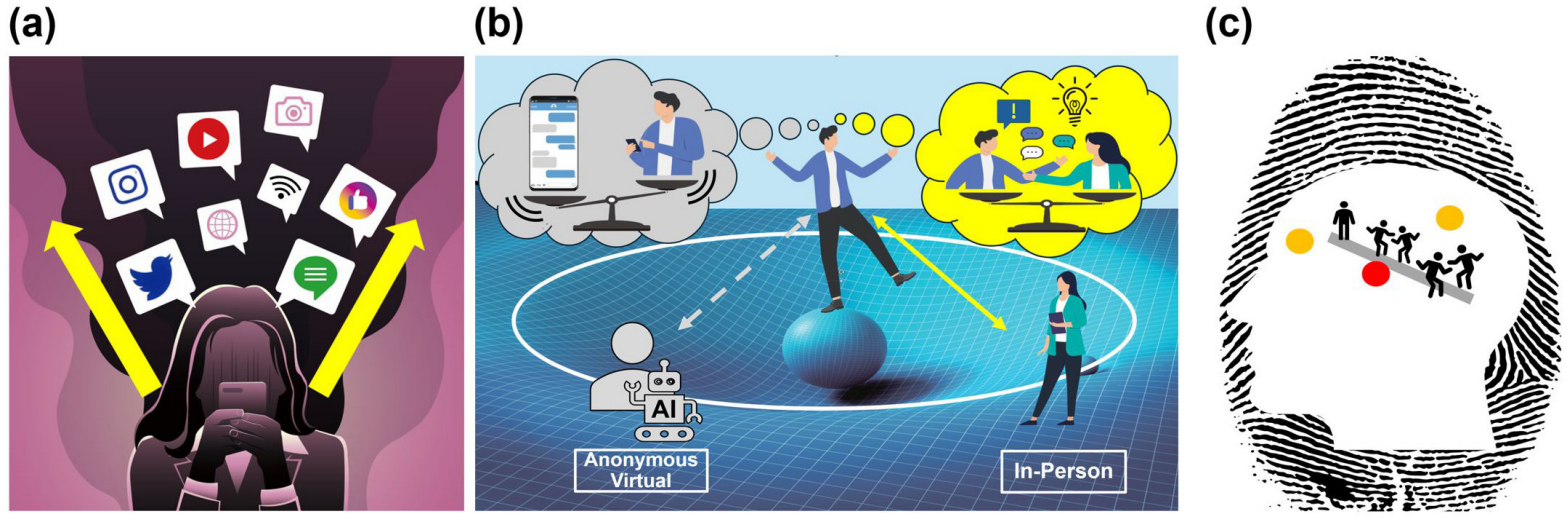
The self in digital communication. **(A)** Self-externalization: Cognitive focus shifts from internal reflection to outward exploration, including emotions, beliefs, and preferences. **(B)** Communication and digital technology: While digital tools and AI-powered agents enrich communication and perspectives, excessive reliance can disrupt interpersonal relationships and the shared center of narrative gravity. **(C)** Self as the center of narrative and cognitive gravity: This figure metaphorically illustrates the self as shaped by the interplay between self and others, along with cognitive processes, drawing on Dennett's (2014) concept of the self as a center of narrative gravity and the DMN's role as the brain's center of gravity for self-processing (Davey and Harrison, 2018). Images are used under license from iStock.

Online self-externalization can alleviate our bodily experience and self-perception by redirecting cognitive focus from internal reflection to outward exploration (Bolis and Schilbach, 2020; Price et al., 2024). Self-externalization enables individuals to project, express, and gain understanding of themselves across online platforms such as health apps and social networking sites (Figure 1A). With a massive online audience, they can create dynamic, context-specific identities that respond to sociocultural norms (Boyd, 2014; Aboujaoude, 2011), offering diverse perspectives (Floridi, 2011) and the potential for disembodiment to reduce social anxiety (McKenna and Bargh, 2000). However, this online self-externalization can also distort emotional regulation and disrupt bodily coherence (Jackson, 2023; Vogel et al., 2015; James et al., 2023).

Online communication and self-externalization can weaken embodied cognition by distancing individuals from their physical, lived experiences (Bolis and Schilbach, 2020), disconnecting the narrative self from bodily awareness. While digital platforms and self-externalization simulate daily activities, they lack the depth of bodily engagement found in real-life encounters (Turkle, 2015; Clark, 2008). Meanwhile, spontaneous interactions in physical spaces, such as supermarkets or bookstores, may offer rich, unexpected, and multisensory experiences—including pleasures, failures, and discomforts—that digital environments often struggle to replicate. These real-world encounters foster a grounded, embodied sense of self and shared narratives, which are often more essential for developing resilience (Gallagher and Zahavi, 2012).

## Discussion

In online environments, the self often emerges as a center of narrative gravity, becoming more dynamic. However, it also becomes more vulnerable to external critique while remaining physically disconnected from others (Turkle, 2011; Agai, 2022; Ashuri et al., 2018). The vast and often anonymous nature of online interactions can reshape our identities and narratives in positive and potentially detrimental ways. Engaging adaptively with online and offline environments enhances people's diversity and flexibility, allowing them to switch between narrative and embodied modes of self depending on the context.

Online communication can enhance self-exploration and perspective renewal but may also fragment the narrative and embodied self (Sleigh et al., 2024; Urzedo et al., 2024). Although online media and AI-powered agents enrich communication, overreliance can undermine interpersonal relationships and the shared center of narrative gravity (Figure 1B). Meanwhile, in-person communication often helps stabilize our narrative core by addressing misalignments (Turkle, 2015; McAdams, 2001), while promoting reciprocal exchanges of thoughts and emotions. The dynamic embodied interactions nurture mutual understanding and bodily coherence, resembling dancers coordinating movements through a shared center of gravity (Supplementary Table S1). This coordination may extend beyond the physical realm into cognitive domains, enhancing collaborative conflict resolution and community dialogue. Moreover, non-verbal communication, a key aspect of in-person interaction, boosts imagination and empathy (Supplementary Table S1), which are essential for handling social tensions and uncertainties. It helps individuals navigate harmony and deviations from shared experiences more adaptively, encouraging deeper connections through in-person communication.

Sharing real-life experiences and building trust also complement virtual interactions, supporting the default mode network (DMN) and its role in self-referential processing (Wang et al., 2017). This network, which is active during introspection and the processing of thoughts and emotions, is sometimes described as the brain's center of gravity for self-processing (Davey and Harrison, 2018). It helps form a worldview through interpersonal experiences and the cohesive forces of identity, aligning with Dennett's (2014) concept of the self as a center of narrative gravity, framing selfhood as a dynamic narrative (Figure 1C). This analogy illustrates that the DMN may serve not only as the center of the self (Qin and Northoff, 2011) but also as a series of nodes and narrative edges that shape interconnected selves (Varela, 1991). Through interpersonal encounters, this process may provide a gravitational pull of identity and intercorporeality.

This collectivistic perspective of the self aligns with autopoiesis theory, highlighting our evolving, relational, and spontaneous nature (Supplementary Table S1). It suggests that our connected and shared identities contribute to a unified sense of self, describing us as dynamic beings who continuously self-produce across physical, experiential, and social levels (Maturana and Varela, 1991; Froese, 2017). Thus, rather than stemming solely from innate qualities, our uniqueness arises from the emergence of selves raised by unrepeatable and unpredictable interpersonal encounters. By responding flexibly and attentively to these conditions (Gallagher et al., 2024; Zhang et al., 2024), we co-construct the narrative and enactive aspects of the self, mind, and life (Di Paolo, 2018).

Balancing technology with real-life connections is essential for navigating narrative identity (Przybylski and Weinstein, 2017; Krueger and Osler, 2019; Kernis and Goldman, 2006). While virtual communication sometimes portrays life as a predetermined path shaped by echo chambers (Sunstein, 2018), real-life interactions resemble rivers—constantly evolving and flowing forward, guided by a naïve gravity toward the Great Sea of Being. In this journey, other people also shape our essence (Bolis et al., 2023), with each path leading to serenity and every choice unfolding new learning opportunities. Adapting to unexpected changes helps our minds flow, refining narratives and fostering tolerance. Marcel Proust's observation that “The real voyage of discovery consists not in seeking new landscapes but in having new eyes” encapsulates the viewpoints on shared understanding and somatic awareness. Although literature and digital content nourish our minds by offering suggestions and simulation, embracing authentic real-life experiences may remain crucial. As Proust further notes, “We don't receive wisdom; we must discover it for ourselves after a journey that no one can take for us or spare us.”

More research is needed to explore the potential of online communication while recognizing the irreplaceable value of face-to-face encounters. The rise of technology and AI increasingly shapes personal perspectives through data-driven viewpoints (Hohenstein et al., 2023). This shift may overshadow individual narratives, altering our sense of self and connection with others. As Plato noted with the invention of writing, each technological advance modulates our perception of self (Floridi, 2011), challenging the essence of what it means to be human (Liberati and Mykhailov, 2024; Fabry, 2023; van Deursen and van Dijk, 2014).

## Conclusion

Digital communication broadens our perspectives in daily life but can also increase online vulnerability, distort self-perception, embodied experiences, and impact public mental health. Overreliance on virtual connections and self-externalization may weaken cohesive identity formation by exposing the core of our narrative gravity to adverse interactions and instability. This can distort self-images and beliefs by shifting cognitive focus away from introspection and seeking outward affirmation. Meanwhile, in-person communication can often complement these effects by grounding us in shared bodily experiences and balanced intercorporeality. This dynamic aligns with autopoietic self-organization. Today, self-poiesis involves a continuous interplay between virtual and physical encounters (Floridi, 2011), reshaping us through online externalization and offline internal reflection, resulting in complex updates to the self. Interconnected and shared identities evolve relationally and spontaneously, synchronizing and diverging in the intricate dance of human precariousness. Continued exploration is essential to address the emerging challenges in navigating the self.
