# Supplementary material for: Navigating the self online
Source: Front Psychol. 2025 Mar 5;16:1499039. doi: 10.3389/fpsyg.2025.1499039 (PMC11919843; doi:10.3389/fpsyg.2025.1499039)
Supplement: Supplementary file 1 [file Table_1.docx]

**Navigating the self online**

**Supplementary Table S1.** Glossary of key terms

| 1. **Mediated and authentic communication**   Mediated communication refers to digital and virtual interactions, while authentic communication involves direct, face-to-face exchanges without digital devices. In authentic communication, individuals engage in the reciprocal exchange of affective experiences, bodily expressions, and non-verbal cues. |
| --- |
| **2. Bodily coherence and gravity center**  Dynamic reciprocal experiences that adjust the centers of gravity of oneself and others foster collaborative movement. For example, activities such as dancing and playing in a musical orchestra illustrate how the active center of gravity continuously reproduces and reorganizes the self in relation to others through attuned interactions and exchanges. |
| **3. Empathy**  Through imagination and empathy, we create shared narratives that shape our beliefs and experiences. These narratives, exchanged within relationships, help form our identities and foster social connections and resilience. However, excessive virtual communication can sometimes weaken imagination and empathy, leading to a disconnect from shared somatic awareness. For instance, constant exposure to mortality figures during the COVID-19 pandemic and recent wars can alter our perception of death. |
| **4. Autopoiesis theory** Varela’s autopoiesis theory, foundational in the study of life and cognition, emphasizes autonomous self-organization (Maturana and Varela, 1991). In multicellular organisms, metabolic and neural activities involve a complex interplay among self-producing cells, which collectively create a unified activation pattern, akin to nodes in neural networks or cells forming organs. Although locally indistinguishable, these components are essential at the organizational level. Varela’s theory of selfless selves (Varela, 1991) extends this concept, depicting the self as a network of interconnected components without a fixed identity. This view portrays the self as relational, spontaneous, and inherently provisional, forming a cohesive whole. Currently, self-poiesis (Floridi, 2011) involves a complex interaction between online and offline communication. |
| 5. Narrative and embodied aspect of self While the narrative self is shaped by past and future experiences, and the embodied self is grounded in the present, flexible and attentive engagement with daily experiences facilitates the adaptive construction of both aspects of the self (Gallagher & Zahavi, 2012; Gallagher et al., 2024). |

# References

Floridi, L. (2011). The informational nature of personal identity. *Minds and Machines*, 21(4), 549-566.

# Gallagher, S., & Zahavi, D. (2012). *The phenomenological mind* (2nd ed.). Routledge.

Gallagher, S., Raffone, A., Berkovich-Ohana, A. *et al.* (2024). The Self-Pattern and Buddhist Psychology. *Mindfulness* 15, 795–803.

Maturana, H.R., Varela, F.J. (1991). *Autopoiesis and cognition: The realization of the living* (Vol. 42). Springer Science & Business Media.

Varela, F.J. (1991). Organism: A meshwork of selfless selves. In *Organism and the Origins of Self* (pp. 79-107). Dordrecht: Springer Netherland.
